# Efficacy and Safety of TransCon PTH in Adults With Hypoparathyroidism: 52-Week Results From the Phase 3 PaTHway Trial

**DOI:** 10.1210/clinem/dgae693

**Published:** 2024-10-08

**Authors:** Bart L Clarke, Aliya A Khan, Mishaela R Rubin, Peter Schwarz, Tamara Vokes, Dolores M Shoback, Claudia Gagnon, Andrea Palermo, Lisa G Abbott, Lorenz C Hofbauer, Lynn Kohlmeier, Filomena Cetani, Susanne Pihl, Xuebei An, Alden R Smith, Bryant Lai, Jenny Ukena, Christopher T Sibley, Aimee D Shu, Lars Rejnmark

**Affiliations:** Endocrinology, Mayo Clinic E18-A, Rochester, MN 55905, USA; Endocrinology, Metabolism, and Geriatrics, McMaster University, Hamilton, ON L8S 4L8, Canada; Endocrinology, Columbia University, New York, NY 10032, USA; Internal Medicine and Endocrinology, Rigshospitalet, 2100 Copenhagen, Denmark; Endocrinology, Diabetes, and Metabolism, University of Chicago, Chicago, IL 60637, USA; Endocrinology, VA Medical Center, University of California, San Francisco, San Francisco, CA 94143, USA; Endocrinology and Nephrology, CHU de Québec-Université Laval Research Centre, Quebec City, QC G1V 4G2, Canada; Department of Medicine, Université Laval, Quebec City, QC G1V 0A6, Canada; Unit of Metabolic Bone and Thyroid Disorders, Fondazione Policlinico Campus Bio-medico, 00128 Rome, Italy; Unit of Endocrinology and Diabetes, Campus Bio-medico University, 00128 Rome, Italy; Endocrinology, Northern Nevada Endocrinology, Reno, NV 89511, USA; Endocrinology, University of Nevada, Reno, Reno, NV 89557, USA; Endocrinology, Diabetes, and Metabolic Bone Diseases, Technische Universität Dresden Medical Center, 01307 Dresden, Germany; Endocrinology and Spokane Osteoporosis, Arthritis Northwest Research Center, Spokane, WA 99223, USA; Endocrine Unit, University Hospital of Pisa, 56126 Pisa, Italy; Clinical Pharmacology and Bioanalysis, Ascendis Pharma A/S, 2900 Hellerup, Denmark; Biostatistics, Ascendis Pharma Inc, Palo Alto, CA 94304, USA; Global Health Economics Outcomes Research, Ascendis Pharma Inc, Palo Alto, CA 94304, USA; Endocrine Medical Sciences, Ascendis Pharma Inc, Palo Alto, CA 94304, USA; Endocrine Medical Sciences, Ascendis Pharma Inc, Palo Alto, CA 94304, USA; Endocrine Medical Sciences, Ascendis Pharma Inc, Palo Alto, CA 94304, USA; Endocrine Medical Sciences, Ascendis Pharma Inc, Palo Alto, CA 94304, USA; Clinical Medicine and Endocrinology, Aarhus University Hospital, 8200 Aarhus N, Denmark

**Keywords:** hypoparathyroidism, parathyroid hormone, hormone replacement therapy, hypocalcemia, palopegteriparatide, quality of life

## Abstract

**Context:**

Conventional therapy for hypoparathyroidism aims to alleviate symptoms of hypocalcemia but does not address insufficient parathyroid hormone (PTH) levels.

**Objective:**

Assess the long-term efficacy and safety of TransCon PTH (palopegteriparatide) for hypoparathyroidism.

**Design:**

Phase 3 trial with a 26-week, double-blind, placebo-controlled period followed by a 156-week, open-label extension (OLE).

**Setting:**

Twenty-one sites across North America and Europe.

**Participants:**

A total of 82 adults with hypoparathyroidism were randomized and received study drug and 78 completed week 52.

**Intervention(s):**

All OLE participants received TransCon PTH administered once daily.

**Main Outcome Measure(s):**

Multicomponent efficacy endpoint: proportion of participants at week 52 who achieved normal serum calcium (8.3-10.6 mg/dL) and independence from conventional therapy (≤600 mg/day of elemental calcium and no active vitamin D). Other efficacy endpoints included patient-reported outcomes and bone mineral density. Safety was assessed by 24-hour urine calcium and treatment-emergent adverse events.

**Results:**

At week 52, 81% (63/78) met the multicomponent efficacy endpoint, 95% (74/78) achieved independence from conventional therapy, and none required active vitamin D. Patient-reported outcomes showed sustained improvements in quality of life, physical functioning, and well-being. Mean bone mineral density *Z*-scores decreased toward age- and sex-matched norms from baseline to week 52. Mean (SD) 24-hour urine calcium excretion decreased from 376 (168) mg/day at baseline to 195 (114) mg/day at week 52. Most treatment-emergent adverse events were mild or moderate and none led to trial discontinuation during the OLE.

**Conclusion:**

At week 52 of the PaTHway trial, TransCon PTH showed sustained efficacy, safety, and tolerability in adults with hypoparathyroidism.

Hypoparathyroidism is an endocrine disease caused by insufficient levels of parathyroid hormone (PTH). Through its direct action on bone and kidney and indirect action on the intestine via increased synthesis of 1,25-dihydroxyvitamin D (calcitriol), PTH is the primary regulator of the calcium-phosphate balance in the body (1, 2). Clinical characteristics of hypoparathyroidism include hypocalcemia, hyperphosphatemia, reduced 1,25 dihydroxyvitamin D levels, elevated fractional excretion of calcium in urine, and low bone turnover relative to age- and sex-matched norms (2-4). As a result, individuals with hypoparathyroidism may experience a range of symptoms including neuromuscular irritability and cognitive impairment as well as renal complications and extraskeletal calcifications (1, 2, 4). Health-related quality of life (HRQoL), physical functioning, and psychological well-being are also negatively impacted in individuals with hypoparathyroidism irrespective of disease etiology or serum calcium levels (5-7).

Conventional therapy for the management of hypoparathyroidism includes active vitamin D (ie, calcitriol or alfacalcidol) and elemental calcium. According to the 2022 Guidelines from the Second International Workshop, treatment with conventional therapy is suggested as first-line therapy to raise serum calcium to the target range (ie, the lower half or just below the reference range) and alleviate symptoms of hypocalcemia while avoiding hypercalciuria to reduce the risk of renal disease (3). However, the Guidelines grade this as a weak recommendation for first-line treatment of hypocalcemia and its symptoms, citing low-quality evidence (3). Though conventional therapy aims to alleviate hypocalcemia and its symptoms, it does not restore normal PTH physiology and is associated with long-term complications including nephrocalcinosis, nephrolithiasis, and renal dysfunction (8). Chronically elevated phosphate levels and a high calcium-phosphate product, resulting from both hypoparathyroidism itself and the long-term use of conventional therapy, can contribute to ectopic calcifications in the kidney, brain, eye, or vasculature (2). Individuals with hypoparathyroidism treated with conventional therapy may report a substantial pill burden (9) with unpredictable fluctuations in serum calcium and symptoms of hypocalcemia, decreased kidney function, reduced HRQoL, impaired physical functioning, and well-being (8, 10). The 2022 Guidelines recommend considering the use of PTH replacement therapy when conventional therapy is deemed unsatisfactory because of inadequate control of biochemical and clinical parameters (ie, symptomatic hypocalcemia, hyperphosphatemia, renal insufficiency, hypercalciuria, and poor quality of life), malabsorption or intolerance of large doses of vitamin D and calcium, or the requirement of high doses of conventional therapy (3). For individuals with hypoparathyroidism, there remains an unmet need for a PTH therapy capable of providing physiological levels of PTH, reducing symptom burden and long-term complications, and improving HRQoL.

TransCon PTH (palopegteriparatide, approved under the brand name YORVIPATH in the European Union and certain other countries as a replacement therapy indicated for the treatment of adults with chronic hypoparathyroidism) is a prodrug of PTH (1-34), administered subcutaneously once daily, with sustained release of active PTH designed to provide PTH levels within the physiological range for 24 hours/day for adults with hypoparathyroidism. TransCon PTH consists of PTH (1-34) bound to an inert methoxypolyethylene glycol carrier via a proprietary TransCon linker. Upon exposure to physiological pH and temperature, autocleavage of the linker occurs and active PTH is released in a sustained manner, leading to a mean PTH exposure within the calculated physiological range over a 24-hour period (11, 12). The phase 3 PaTHway trial of TransCon PTH in adults with chronic hypoparathyroidism met the primary and all key secondary endpoints with statistically significant differences from placebo (13). In the 26-week blinded period of the pivotal trial, significantly more participants treated with TransCon PTH (n = 48/61, 79%) than placebo (n = 1/21, 5%) met the primary multicomponent efficacy endpoint, defined as normal serum calcium and independence from conventional therapy without an increase in prescribed study drug over 4 weeks before week 26 (*P* < .0001). Treatment with TransCon PTH was well-tolerated, normalized mean 24-hour urine calcium excretion, and improved HRQoL, disease-specific symptoms, physical functioning, and overall well-being (13). This analysis assesses the long-term efficacy, safety, and tolerability of TransCon PTH for hypoparathyroidism in the ongoing open-label extension (OLE) of the PaTHway trial through week 52.

## Materials and Methods

### Trial Design

The PaTHway trial design was previously published in detail (13). Briefly, PaTHway is a phase 3 multicenter, randomized trial with a 26-week placebo-controlled, parallel-group, double-blind period and an ongoing 156-week OLE designed to investigate the efficacy, safety, and tolerability of TransCon PTH administered once daily in adults with hypoparathyroidism. Participants were men and nonpregnant women (aged ≥18 years) with chronic hypoparathyroidism for a duration of at least 6 months, and included individuals with postsurgical, autoimmune, genetic, and idiopathic etiologies. Additional inclusion and exclusion criteria are published elsewhere (13). At baseline, participants were prescribed once-daily TransCon PTH (18 μg PTH (1-34)/day) or a corresponding volume of placebo and were individually and progressively titrated to an optimal dose (allowable range, 6-60 μg/day) in increments or decrements of 3 μg/day. All treatment injections were administered by a Ypsomed UnoPen Fix pen injector using 31-gauge, 5-mm pen needles to deliver doses of 6 to 30 μg/injection in a volume of ≤100 μL to either abdomen or anterior thigh, rotating injection sites. Following successful completion of the 26-week blinded treatment period, participants were eligible to enter the OLE where all received TransCon PTH. At the week 26 visit, all participants randomized to placebo during the blinded period were started on TransCon PTH (18 μg PTH (1-34)/day) in addition to conventional therapy and were individually and progressively titrated to an optimal dose according to a protocol-specified algorithm guided by serum calcium values (13). The protocol was reviewed by the appropriate institutional review boards and independent ethics committees (ClinicalTrials.gov identifier: NCT04701203; EudraCT No.: 2020-003380-26). The trial was designed by the sponsor and authors and conducted in accordance with the principles of the Declaration of Helsinki and Good Clinical Practice guidelines as described in the International Conference on Harmonization Guideline E6. Results through week 52 of the PaTHway trial are reported here.

### Efficacy Assessments

In the PaTHway OLE, TransCon PTH efficacy was assessed by a multicomponent endpoint defined as the proportion of participants who achieved albumin-adjusted serum calcium within the normal range (8.3-10.6 mg/dL [2.07-2.64 mmol/L]), independence from active vitamin D (defined as a standing dose of active vitamin D equal to zero on the day before the visit of interest), and independence from therapeutic doses of calcium (defined as a standing dose of elemental calcium ≤600 mg on the day before the visit of interest). Other efficacy endpoints evaluated at predefined time points during the OLE included active vitamin D and calcium doses, daily pill burden of active vitamin D and calcium (as oral tablets, powder, liquid solutions, liquid suspensions, or transdermal patches), albumin-adjusted serum calcium, serum phosphate, and albumin-adjusted serum calcium × phosphate product.

Two patient-reported outcome (PRO) measures, the Hypoparathyroidism Patient Experience Scale (HPES) and the 36-Item Short-From Health Survey (SF-36), were collected at baseline and weeks 10, 20, 26, 34, and 52. The HPES is a psychometrically validated, disease-specific measure designed to assess hypoparathyroidism-specific symptoms, functioning, and well-being from the patient perspective. The 17-item HPES-Symptom assesses key physical and cognitive symptoms, whereas the 26-item HPES-Impact assesses the impact of these symptoms on physical functioning, daily life, psychological well-being, social life, and relationships (6, 7). Domain and total scores range from 0 to 100, with higher scores indicating greater disease impact. The SF-36 is a validated, generic HRQoL questionnaire with 8 subscales (physical functioning, role physical, bodily pain, general health, vitality, social functioning, role emotional, and mental health) and physical and mental component summary scores (14). SF-36 scores are expressed relative to the average of the general US population, with higher scores indicating better HRQoL. In the blinded treatment period, the change from baseline to week 26 in HPES-Symptom physical and cognitive domain scores and HPES-Impact physical functioning and daily life domain scores; the SF-36 physical functioning subscale were key secondary endpoints. Key secondary endpoint PRO domains are included in the week 52 analysis.

Indices of skeletal dynamics, including bone mineral density (BMD) and bone turnover markers (BTMs), were also evaluated in the PaTHway trial. Bone mineral density at 4 regions of interest (lumbar spine [L1-L4], total hip, femoral neck, and distal 1/3 radius) was measured by dual-energy x-ray absorptiometry at baseline, week 26, and week 52. Observed BMD values in g/cm^2^ were transformed into T-scores and *Z*-scores. Serum levels of the bone formation biomarker procollagen type 1 N-terminal propeptide (P1NP) and bone resorption biomarker C-terminal telopeptide of type 1 collagen (CTx) were measured at baseline and weeks 12, 26, 38, and 52.

### Safety Assessments

Safety parameters collected during blinded treatment and the OLE included serum chemistries, 24-hour urine chemistry (including 24-hour urine calcium excretion), hematology, 1,25-dihydroxyvitamin D levels, and antibodies against PTH, TransCon PTH (prodrug), and polyethylene glycol (PEG). Treatment-emergent adverse events (TEAEs), treatment-emergent serious adverse events (SAEs), and adverse events of special interest were documented by site staff at clinic visits. Trial investigators evaluated the seriousness, severity, and causality of all reported TEAEs. Adverse events were coded by system organ class and preferred term using the Medical Dictionary for Regulatory Activities Version 24.0 or newer. The World Health Organization toxicity grading scale was used for assessing TEAE severity (grade 1 = mild, grade 2 = moderate, grade 3 = severe, grade 4 = life-threatening).

### Statistical Analysis

Data are summarized descriptively by treatment group and for total participants with continuous variables presented as mean and SD or median and range or interquartile range (IQR), and categorical data presented using counts (n) and percentages of participants. At post-baseline visits, only data from participants with both baseline and the corresponding visit values were used to compute statistical summaries. The period of exposure to TransCon PTH in OLE analyses is defined as the time from exposure to the first dose of TransCon PTH until the time of analysis. For participants randomized to TransCon PTH at trial enrollment, this is the time from exposure to the first dose of blinded TransCon PTH to the time of analysis. For participants randomized to placebo at enrollment, it is the time from exposure to the first open-label dose of TransCon PTH to the time of analysis. All CIs are 2-sided 95% CIs unless stated otherwise. All statistical analyses were conducted using SAS version 9.4.

## Results

### Participant Disposition and Treatment Exposure

**Figure 1.**
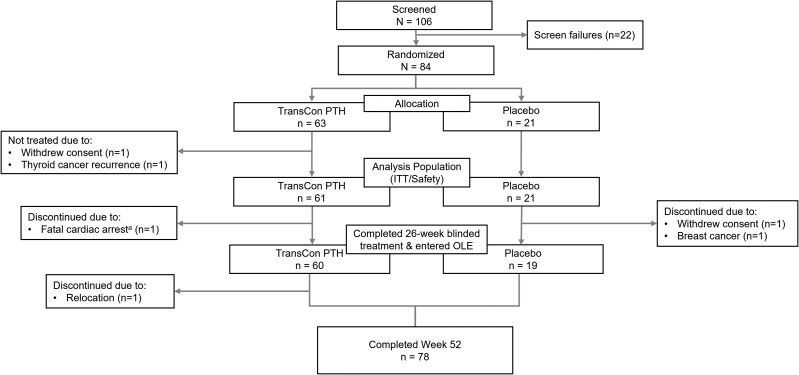
PaTHway trial participant disposition through week 52. A total of 106 participants were screened, 84 met eligibility criteria, and 82 were enrolled in the trial and were randomized to treatment. The intent-to-treat (ITT) analysis population comprised 61 participants in the TransCon PTH and 21 participants in the placebo group who received ≥1 blinded treatment. ^a^Fatal cardiac arrest deemed unrelated to study drug. OLE, open-label extension.

In the PaTHway trial, 82 participants were randomized to treatment and received at least 1 dose of blinded study drug (n = 61 TransCon PTH, n = 21 placebo), and 79 participants (n = 60 TransCon PTH, n = 19 placebo) completed blinded treatment through week 26 and entered the OLE. Of those participants, 78 (n = 59 TransCon PTH/TransCon PTH, n = 19 placebo/TransCon PTH) completed week 52 (Fig. 1). One participant withdrew consent during the OLE. The PaTHway trial population was predominantly female (n = 64/82, 78%) and White (n = 76/82, 93%) with a mean (SD) baseline age of 48.6 (12.7) years. Thirty-four percent (n = 22/64) of female participants were postmenopausal at baseline. The mean (SD) duration of hypoparathyroidism at baseline was 11.7 (10.7) years. The majority of participants (n = 70/82, 85%) had postsurgical hypoparathyroidism. Other etiologies included primary idiopathic disease (n = 7), autoimmune polyglandular syndrome type 1 (n = 2), autosomal dominant hypocalcemia (activating mutation of the calcium-sensing receptor) (n = 1), hypoparathyroidism, sensorineural deafness, and renal disease (also known as Barakat syndrome) (n = 1), and DiGeorge syndrome (n = 1). At week 52, the mean (SD) daily TransCon PTH dose was 24.6 (7.6) μg/day (median 24.0 μg/day, range 9-54 μg/day). Mean (SD) treatment compliance was 97.1 (3.4)%, defined as the total number of actual TransCon PTH doses divided by the total number of planned doses expressed as a percentage.

### Efficacy

**Figure 2.**
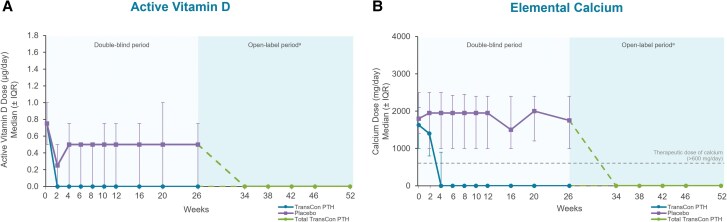
Conventional therapy doses through week 52 of the PaTHway trial. (A) Median (IQR) active vitamin D dose (µg/day). At week 52, none of the participants were taking active vitamin D daily. (B) Median (IQR) elemental calcium dose (mg/day). Per trial protocol, participants were permitted to take calcium ≤600 mg/day as a nutritional supplement, if needed, to meet the recommended dietary intake of calcium. ^a^Participants randomized to placebo during the blinded period initiated TransCon PTH treatment at week 26. Data are shown separately by randomized treatment allocation through week 26, after which all participants received TransCon PTH during the open-label period (dashed lines). Data are combined for all participants from week 34 through 52. IQR, interquartile range.

**Figure 3.**
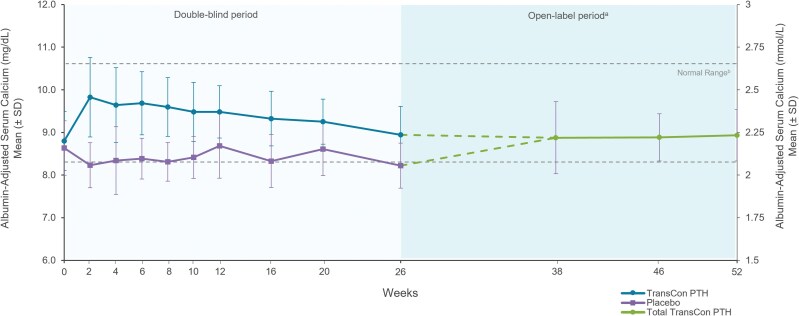
Albumin-adjusted serum calcium with TransCon PTH treatment through week 52. Albumin-adjusted serum calcium levels were maintained within the normal range at all time points in the OLE for all participants, with a mean of 8.9 mg/dL at week 52. ^a^Participants randomized to placebo during the blinded period initiated TransCon PTH treatment at week 26. Data are shown separately by randomized treatment allocation through week 26, after which all participants received TransCon PTH during the open-label period (dashed lines). Data are combined for all participants from week 38 through 52. ^b^Normal range for serum calcium = 8.3-10.6 mg/dL (2.07-2.64 mmol/L).

At week 52 of the PaTHway trial, 81% (n = 63/78) of participants treated with TransCon PTH met the multicomponent efficacy endpoint and 95% (n = 74/78) achieved independence from conventional therapy (Table 1). The mean elemental calcium dose at week 52 was 148 (418) mg/day (median [range] 0 [0, 3000]) and none of the participants were taking active vitamin D. Treatment with TransCon PTH enabled rapid and sustained reduction of elemental calcium doses (Fig. 2A) and complete discontinuation of active vitamin D for those randomized to placebo at baseline and transitioned to open-label TransCon PTH at week 26 (Fig. 2B), consistent with results from the TransCon PTH group during the blinded period. At week 52, 61 participants were taking no elemental calcium, 13 were taking a dose ≤600 mg/day (median [range] 480 [214, 600]), and 4 were taking a dose >600 mg/day (median [range] 1200 [900, 3000]). In participants randomized to the TransCon PTH group, mean (SD) daily pill burden decreased from 6.7 (2.2) at baseline to 0.45 (1.7) at week 26 and further decreased to 0.26 (0.7) at week 52. Mean (SD) daily pill burden in the placebo group was 6.7 (3.0) at baseline and 5.4 (3.2) at week 26. With the initiation of TransCon PTH treatment at week 26, pill burden decreased to a mean (SD) of 0.5 (0.8) at week 52 for this group. Mean albumin-adjusted serum calcium levels were maintained within the normal range at all time points during open-label TransCon PTH treatment (Fig. 3). In those randomized to placebo during blinded treatment, mean (SD) albumin-adjusted serum calcium levels increased from 8.2 (0.5) mg/dL (2.05 [0.12] mmol/L) at week 26 to within the normal range with the initiation of TransCon PTH treatment and were maintained through week 52 (9.1 [0.6] mg/dL; 2.27 [0.15] mmol/L)]. Mean serum phosphate, calcium × phosphate product, magnesium, 25-hydroxyvitamin D, and 1,25-dihydroxyvitamin D values were maintained within normal ranges throughout the OLE (Table 2).

**Table 1.** PaTHway trial open-label extension multi-component efficacy endpoint at week 52

	Total TransCon PTH (N = 82)
Participants with data on all criteria at week 52, n	78
Participants meeting the multi-component endpoint at week 52 (responders), n	63
Proportion, % (95% CI)*^a^*	81 (70, 89)
Number of participants meeting each component, n (%)	
Albumin-adjusted serum calcium within the normal range*^b^*	67 (86)
Independence from active vitamin D*^c^*	78 (100)
Independence from therapeutic doses of calcium*^d^*	74 (95)

Abbreviations: ITT, intent to treat.

^*a*^Percentages are calculated based on ITT participants who had data on all criteria.

^*b*^Normocalcemia defined as 8.3 to 10.6 mg/dL.

^*c*^Independence defined as a standing dose of active vitamin D equal to zero on the day before the week 52 visit.

^*d*^Independence defined as a standing dose of elemental calcium ≤600 mg on the day before the week 52 visit.

**Table 2.** Serum biochemistries through week 52 of the PaTHway trial

	Baseline		Week 26		Week 52
Serum biochemistries	TransCon PTH (N = 61)	Placebo (N = 21)	TransCon PTH (N = 60)	Placebo (N = 19)	Total TransCon PTH (N = 78)
Albumin-adjusted calcium (mg/dL)					
Mean (SD)	8.8 (0.7)	8.6 (0.6)	8.9 (0.7)	8.2 (0.5)	8.9 (0.6)
Median (Q1, Q3)	8.8 (8.4, 9.2)	8.5 (8.3, 9.1)	9.0 (8.6, 9.3)	8.4 (7.9, 8.6)	8.9 (8.6, 9.3)
Phosphate (mg/dL)					
Mean (SD)	4.2 (0.6)	3.9 (0.8)	3.8 (0.6)	3.9 (0.9)	3.7 (0.7)
Median (Q1, Q3)	4.2 (3.9, 4.6)	3.7 (3.2, 4.6)	4.2 (3.9, 4.6)	3.7 (3.2, 4.7)	3.7 (3.3, 4.0)
Calcium*^a^* × phosphate product (mg^2^/dL^2^)					
Mean (SD)	37.1 (5.7)	33.7 (6.7)	33.9 (4.8)	31.7 (6.4)	32.8 (5.9)
Median (Q1, Q3)	37.5 (33.5, 40.0)	33.1 (27.1, 40.5)	34.0 (30.4, 36.8)	32.6 (27.3, 35.7)	33.7 (28.2, 35.7)
Magnesium (mg/dL)					
Mean (SD)	2.1 (0.2)	2.0 (0.2)	2.2 (0.1)	2.0 (0.2)	2.1 (0.2)
Median (Q1, Q3)	2.1 (2.0, 2.2)	2.0 (1.9, 2.1)	2.1 (1.1, 2.2)	2.1 (1.8, 2.1)	2.1 (2.0, 2.2)
1,25-Dihydroxyvitamin D (pg/mL)*^b^*					
Mean (SD)	38.5 (11.0)	40.0 (15.4)	39.6 (19.3)	34.8 (16.5)	41.5 (16.2)
Median (Q1, Q3)	39.3 (30.7, 44.6)	38.0 (28.5, 44.6)	38.2 (25.7, 51.5)	33.7 (27.0, 41.9)	41.2 (29.6, 47.9)
25-Hydroxyvitamin D (ng/mL)*^b^*					
Mean (SD)	43.2 (11.9)	41.1 (11.5)	36.4 (12.0)	42.2 (17.4)	39.0 (13.6)
Median (Q1, Q3)	42.5 (35.3, 50.5)	38.8 (32.5, 45.7)	34.5 (28.0, 41.3)	38.1 (32.9, 46.9)	37.2 (29.6, 42.9)
eGFR (mL/min/1.73 m^2^)*^c^*					
Mean (SD)	67.3 (137)	72.7 (14.6)	75.3 (14.4)	70.8 (12.4)	77.4 (15.4)
Median (Q1, Q3)	66.6 (57.8, 76.5)	71.7 (65.3, 76.9)	75.7 (66.3, 84.8)	74.7 (61.8, 81.0)	77.2 (67.8, 88.2)

Abbreviations: eGFR, estimated glomerular filtration rate; Q, quartile.

^*a*^Albumin-adjusted serum calcium.

^*b*^n = 59 for TransCon PTH group at week 26.

^*c*^eGFR adjusted for body surface area.

### Patient-Reported Outcomes

**Figure 4.**
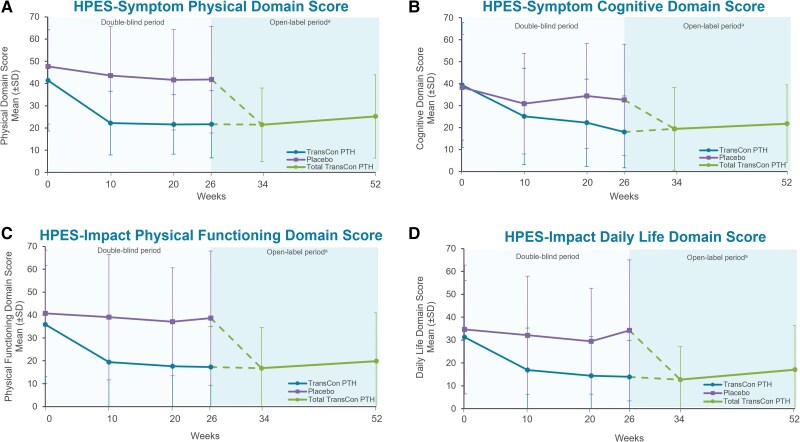
Long-term effect of TransCon PTH on participant-reported symptom burden and impact. The Hypoparathyroidism Patient Experience Scale (HPES) scores showed sustained improvements in hypoparathyroidism-related physical and cognitive symptoms and the impact of disease-specific symptoms on physical functioning and daily life with TransCon treatment at week 52. In participants first treated with placebo, HPES scores from weeks 26 to 52 showed the same rapid improvement as seen in participants treated with TransCon PTH during the blinded period. Higher HPES scores are associated with greater disease burden. ^a^Participants randomized to placebo during the blinded period initiated TransCon PTH treatment at week 26. Data are shown separately by randomized treatment allocation through week 26, after which all participants received TransCon PTH during the open-label period (dashed lines). Data are combined for all participants from week 34 through 52. Negative error bars (SD) are not displayed for values less than zero.

In participants randomized to TransCon PTH at baseline, HPES scores showed sustained improvements in hypoparathyroidism-related symptoms, functioning, and well-being through week 52. The mean (SD) changes from baseline to week 52 in these participants were −17.4 (17.3) in HPES-Symptom physical domain scores, −19.1 (24.0) in HPES-Symptom cognitive domains scores, −16.0 (18.4) in HPES-Impact physical functioning domain scores, and −15.2 (18.2) in HPES-Impact daily life domain scores. With the initiation of TransCon PTH treatment at week 26, HPES scores rapidly improved in those previously treated with placebo, similar to the active treatment group during the blinded period (Fig. 4). SF-36 subscale and component summary scores also remained above baseline and showed a sustained improvement from baseline in HRQoL with TransCon PTH treatment over 52 weeks. In participants randomized to placebo, TransCon PTH treatment in the OLE was associated with improvements in SF-36 scores similar to those observed in the active treatment group during the blinded period.

### Indices of Skeletal Dynamics

**Figure 5.**
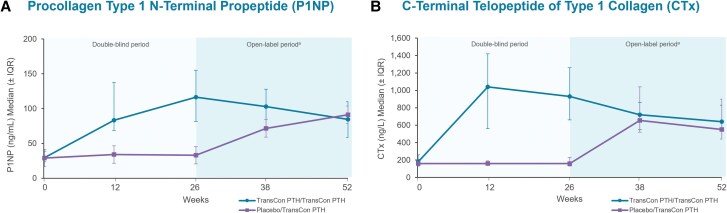
Bone turnover biomarkers through week 52. (A) Serum procollagen type 1 N-terminal propeptide (P1NP) in TransCon PTH/TransCon PTH participants increased from a median of 29.4 ng/mL at baseline to a peak of 116.5 ng/mL at week 26 and was 84.4 ng/mL at week 52. (B) In TransCon PTH/TransCon PTH participants serum C-terminal telopeptide of type 1 collagen (CTx) (baseline median 180.0 ng/L) peaked at 1040.0 ng/L at week 12 and was 640.0 ng/L at week 52. Normal serum biomarker ranges: P1NP (ng/mL) premenopausal women 15 to 59; postmenopausal women 16 to 74; men 14 to 86 and CTx (ng/L) premenopausal women 30 to 570; postmenopausal women 100 to 1010; men 18 to 30 years: 160 to 870; men 31 to 50 years: 90 to 630; men ≥51 years: 40 to 840. ^a^Participants randomized to placebo during the blinded period initiated TransCon PTH treatment at week 26. IQR, interquartile range.

In participants treated with TransCon PTH in both the blinded and open-label periods, smaller incremental changes were generally seen in BTMs and BMD between week 26 and week 52 than baseline and week 26. The bone formation biomarker P1NP increased from a median (IQR) of 29.4 (23.7, 39.0) ng/mL at baseline to a peak of 116.5 (81.4, 155.3) ng/mL at week 26 and trended down to 84.4 (58.6, 110.0) ng/mL at week 52 (Fig. 5A). Median (IQR) CTx (bone resorption biomarker) levels were 180.0 (140.0, 260.0) ng/L at baseline, peaked at 1040.0 (560.0, 1420.0) ng/L at week 12, and were 640.0 (440.0, 830.0) ng/L at week 52 (Fig. 5B). In participants randomized to placebo at baseline, trends in BTMs with TransCon PTH treatment from week 26 to week 52 resembled those observed in the TransCon PTH group from baseline to week 26. Bone mineral density by dual-energy x-ray absorptiometry, T-scores, and *Z*-scores at baseline, week 26, and week 52 in participants randomized to TransCon PTH at baseline are shown in Table 3. Mean BMD T-scores and *Z*-scores in these participants were within the normal range at all sites at week 52. At the lumbar spine, total hip, and femoral neck, the percent change in BMD in participants randomized to TransCon PTH at baseline was −8.1%, −6.5%, and −7.0% from baseline to week 26, and −0.6%, −1.5%, and −1.5% from week 26 to 52, respectively. At the distal 1/3 radius, the percent change in BMD was −0.06% from baseline to week 26 and −0.5% from week 26 to week 52.

**Table 3.** Bone mineral density by dual-energy x-ray absorptiometry T-scores and *Z*-scores with TransCon PTH at baseline, week 26, and week 52

	TransCon PTH/TransCon PTH (N = 61)		
Location/region parameter (mean)	Baseline*^a^*	Week 26*^b^*	Week 52*^b^*
Spine/adjusted total (L1-L4)			
n	59	58	57
BMD (g/cm^2^)	1.20	1.11	1.10
T-score	0.92	0.07	0.02
*Z*-score	1.51	0.68	0.66
Hip/femoral neck			
n	60	59	58
BMD (g/cm^2^)	0.94	0.88	0.86
T-score	0.00	−0.47	−0.58
*Z*-score	0.80	0.34	0.25
Hip/total			
n	60	59	58
BMD (g/cm^2^)	1.04	0.98	0.96
T-score	0.43	−0.08	−0.19
*Z*-score	0.95	0.45	0.36
Forearm/radius 1/3 distal			
n	60	60	59
BMD (g/cm^2^)	0.77	0.76	0.76
T-score	−0.33	−0.34	−0.40
*Z*-score*^c^*	0.33	0.35	0.33

Abbreviation: BMD, bone mineral density.

^*a*^Statistical summaries are computed from all subjects with data at baseline.

^*b*^At postbaseline visits, only data from subjects with both baseline and the corresponding visit values available were used to compute the statistical summaries.

^*c*^n = 59 at baseline and week 26; n = 58 at week 52.

### Safety

**Figure 6.**
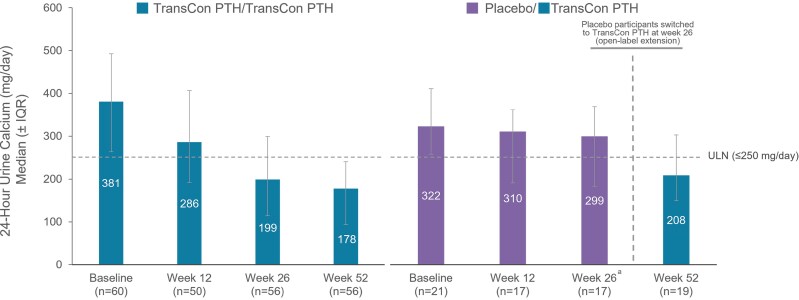
24-Hour urine calcium through week 52. At week 52 of the PaTHway trial, overall median (IQR) 24-hour urine calcium excretion with TransCon PTH was 188.0 (103, 254) mg/day. ^a^Participants randomized to placebo during the blinded period initiated TransCon PTH treatment at week 26.

In participants randomized to TransCon PTH at baseline, 24-hour urine calcium excretion remained below the upper limit of normal (≤250 mg/day) with a mean (SD) of 185.1 (120.8) mg/day at week 52. In the placebo group, mean (SD) 24-hour urine calcium excretion decreased from 292.5 (125.5) mg/day at week 26 to 223.1 (89.3) mg/day at week 52 with TransCon PTH (Fig. 6). Overall, 72 participants treated with TransCon PTH reported TEAEs and 8 reported SAEs (Table 4). Most TEAEs were grade 1 (34% mild) or grade 2 (46% moderate) in severity and none led to treatment discontinuation or trial withdrawal during the OLE. Of the 80 participants treated with TransCon PTH, TEAEs reported by 42 participants (53%) and SAEs reported by 2 participants (3%) were deemed related to treatment. Treatment-related TEAEs reported by ≥5% of participants included injection site reactions (n = 21, 26%), hypercalcemia (n = 11, 14%), nausea (n = 7, 9%), headache (n = 6, 8%), and hypocalcemia (n = 4, 5%). Adverse events of hypercalcemia occurred exclusively in the first 90 days after initiation of treatment, concurrent with the most active phase of titration. Both treatment-related SAEs were due to hypercalcemia and occurred in the setting of inadvertent deviations from the titration algorithm. Both events resolved with supportive therapy, and study treatment was thereafter resumed.

**Table 4.** Treatment-emergent adverse events through week 52 of the PaTHway trial

Treatment-emergent adverse events (TEAEs), n (%)	TransCon PTH/TransCon PTH (n = 61)	Placebo/TransCon PTH*^a^* (n = 19)	Total TransCon PTH (N = 80)
Any TEAE	56 (91.8)	16 (84.2)	72 (90.0)
Serious TEAE	5 (8.2)	3 (15.8)	8 (10.0)
Severity*^b,c^*			
Grade 1	26 (42.6)	11 (57.9)	37 (46.3)
Grade 2	26 (42.6)	1 (5.3)	27 (33.8)
Grade 3	3 (4.9)	4 (21.1)	7 (8.8)
Grade 4*^d^*	1 (1.6)	0	1 (1.3)
Related TEAE	33 (54.1)	9 (47.4)	42 (52.5)
Serious related TEAE	1 (1.6)	1 (5.3)	2 (2.5)
TEAE related to hyper- or hypocalcemia leading to emergency department/urgent care visit and/or hospitalization	4 (6.6)	2 (10.5)	6 (7.5)
TEAE leading to discontinuation of study drug*^d^*	1 (1.6)	0	1 (1.3)
TEAE leading to death*^d^*	1 (1.6)	0	1 (1.3)

^*a*^Includes only TEAEs occurring on or after the first dose of TransCon PTH.

^*b*^Participants are displayed for the highest severity category only.

^*c*^The World Health toxicity grading scale was used to assess TEAE severity; grade 1 = mild, grade 2 = moderate; grade 3 = severe; grade 4 = life-threatening.

^*d*^Death from cardiac arrest in 1 participant during the blinded period that was deemed unrelated to study drug.

No anti-PTH antibodies were detected. Anti-PEG and anti-TransCon PTH antibodies were detected in 17% and 9% of participants at baseline, respectively. A treatment-induced response was observed in 8% and 6% of participants for anti-PEG and anti-TransCon PTH antibodies, respectively, during the 52-week dosing period. When anti-TransCon PTH antibodies were detected, anti-PEG antibodies were also detected in the majority of these samples, suggesting PEG as the epitope for anti-TransCon PTH antibodies. None of the antibodies were neutralizing and the presence of antibodies had no apparent impact on efficacy or safety outcomes.

## Discussion

Long-term treatment with TransCon PTH through week 52 of the phase 3 PaTHway trial showed a durable and favorable efficacy, safety, and tolerability profile in adults with hypoparathyroidism. Seventy-eight participants (98%) who completed the blinded period and continued into the OLE remained on TransCon PTH treatment through week 52, and no participant discontinued treatment because of loss of efficacy or an adverse event. Overall, 81% of participants treated with TransCon PTH achieved independence from conventional therapy while maintaining normocalcemia. In addition to sustained improvements from baseline in 24-hour urine calcium excretion and patient-reported measures of disease burden and HRQoL, indices of skeletal dynamics trended toward age- and sex-matched norms with TransCon PTH treatment. Participants randomized to placebo in the blinded treatment period showed rapid improvements in clinical and PROs after initiation of open-label TransCon PTH treatment, consistent with the results of the active treatment group during the blinded period (13). These results strengthen the growing evidence base demonstrating the ability of TransCon PTH to restore the physiological functions of PTH in adults with hypoparathyroidism.

In the 26-week blinded period of the PaTHway trial, 79% (n = 48/61) of participants treated with TransCon PTH compared to 5% (n = 1/21) treated with placebo achieved the primary multicomponent efficacy endpoint: normal serum calcium and independence from conventional therapy, without an increase in prescribed study drug over 4 weeks before week 26 (*P* < .0001) (13). Treatment with TransCon PTH over 52 weeks maintained complete independence from active vitamin D in all participants and independence from both active vitamin D and therapeutic doses of elemental calcium in 95% of participants. Both the HPES and SF-36 scores also showed sustained improvements from baseline in hypoparathyroidism-related symptoms and their impact on physical functioning and daily life as well as in HRQoL, respectively. These findings support and reinforce the long-term results of the ongoing phase 2 PaTH Forward trial, in which 93% of participants achieved independence from conventional therapy through 110 weeks (15) and improvements in PROs were sustained throughout the OLE (16).

Hypoparathyroidism is associated with notable skeletal abnormalities (3, 17), including reduced bone turnover that leads to high bone density relative to age- and sex-matched controls (2). Conventional therapy for hypoparathyroidism (active vitamin D and calcium), targets hypocalcemia but does not restore the physiological effects of PTH on bone (17). Indices of skeletal dynamics through week 52 of the PaTHway trial suggest that TransCon PTH augments the turnover of bone in a low turnover state and mobilizes calcium from the abnormally dense skeleton of adults with hypoparathyroidism. In participants treated with TransCon PTH during the blinded period and OLE, mean bone resorption biomarker serum CTx levels peaked at week 12 and bone formation biomarker serum P1NP levels peaked at week 26. Similar results were seen through week 110 of the phase 2 PaTH Forward trial, in which BTMs increased from the low end of normal at baseline, peaked in the initial weeks, and trended downward toward age- and sex-matched norms through week 110 of TransCon PTH treatment. As a result of calcium mobilization out of an abnormally dense and hypodynamic bone in response to TransCon PTH, mean BMD *Z*-scores trended toward age- and sex-matched norms and remained above zero for all regions of interest through week 52. Additionally, mean T-scores remained in the normal range. Smaller incremental changes were seen in BMD and BTMs between weeks 26 and 52 than from baseline to week 26. Treatment of hypoparathyroidism with TransCon PTH in long-term phase 2 and 3 clinical trials demonstrates a consistent and sustained trend toward a new skeletal steady state closer to age-appropriate norms (15).

TransCon PTH continued to be generally well-tolerated over 52 weeks in the PaTHway trial with a safety profile similar to that of the 26-week blinded period. Treatment-emergent AEs in the PaTHway trial were mostly mild or moderate in severity, and no treatment-related TEAEs led to trial discontinuation. Mean 24-hour urine calcium excretion remained within normal limits for participants randomized to TransCon PTH treatment at baseline and decreased from >250 mg/day at week 26 to within the normal range by week 52 in those who received TransCon PTH during the OLE only. In contrast, conventional therapy does not restore the calcium-retaining effect of PTH in the kidney and is associated with iatrogenic complications resulting from hypercalciuria and consequent long-term complications including nephrocalcinosis, nephrolithiasis, and renal dysfunction (8).

Results through week 52 of the PaTHway trial confirm and extend previously published clinical trial data that demonstrate the ability of TransCon PTH to restore functions of PTH including the endogenous production of calcitriol and mobilization of calcium from the skeleton and reduce the symptom burden associated with hypoparathyroidism and improve physical functioning and well-being (13, 18, 19). The unique design of TransCon PTH provides sustained release of active PTH and continuous PTH exposure within the physiological range over the 24-hour dosing period with a low peak-to-trough ratio (12), which may contribute to these observed benefits.

Strengths of this trial include a participant population representative of the broader international community of adults with hypoparathyroidism, an excellent participant retention rate in the PaTHway OLE, and the prospective nature of the trial. Limitations of this trial warrant consideration. Investigators and participants were aware of doses of both open-label study drug and conventional therapy during weeks 27 through 52. This may impact the assessment of PROs. However, the first 26 weeks of treatment with TransCon PTH in participants randomized to placebo in the blinded period showed rapid improvement in clinical and PRO endpoints consistent with the pattern in the active treatment group during the 26-week blinded period. Because normative data for the SF-36 are not available for all participating countries in the trial, the US default normative data were used (14), potentially limiting the interpretation of the normative scoring. Normalization of 24-hour urinary calcium excretion, though a target of therapy in clinical practice (3), is a surrogate endpoint. Additional studies are necessary to determine the impact of TransCon PTH on events such as nephrocalcinosis and the development of renal dysfunction.

Treatment of adults with chronic hypoparathyroidism with TransCon PTH over 52 weeks in the ongoing PaTHway trial demonstrated durable efficacy and safety with evidence of physiological functions of PTH in the bone and kidney. TransCon PTH treatment was associated with sustained improvements in clinical outcomes as well as disease-related symptoms and the impact of hypoparathyroidism on physical functioning and daily life. These results suggest that TransCon PTH may improve outcomes and advance the standard of care for adults living with hypoparathyroidism.

## Data Availability

The datasets generated during and/or analyzed during the current study are not publicly available but are available from the corresponding author upon reasonable request.

## Clinical Trial Information

ClinicalTrials.Gov, Number: NCT04701203.

## Abbreviations

BMD: bone mineral density
BTM: bone turnover marker
CTx: type 1 collagen
HPES: Hypoparathyroidism Patient Experience Scale
HRQoL: health-related quality of life
IQR: interquartile range
OLE: open-label extension
P1NP: procollagen type 1 N-terminal propeptide
PEG: polyethylene glycol
PRO: patient-reported outcome
SAE: serious adverse event
SF-36: Short Form-36
TEAE: treatment-emergent adverse event
