# Correction to: “Efficacy and Safety of TransCon PTH in Adults With Hypoparathyroidism: 52-Week Results From the Phase 3 PaTHway Trial”

**DOI:** 10.1210/clinem/dgaf048

**Published:** 2025-02-04

In the above-named article by Clarke BL, Khan AA, Rubin MR, Schwarz P, Vokes T, Shoback DM, Gagnon C, Palermo A, Abbott LG, Hofbauer LC, Kohlmeier L, Cetani F, Pihl S, An X, Smith AR, Lai B, Ukena J, Sibley CT, Shu AD, and Rejnmark L (*J Clin Endo Metab*. 2024; doi: 10.1210/clinem/dgae693), there were errors in Figure 6.

In the originally published article, in the legend of Figure 6, the first sentence read “24-Hour urine calcium through week 52 at week 52 of the PaTHway trial, overall median (IQR) 24-hour urine calcium excretion with TransCon PTH was 188.0 (103, 254) mg/day.” This sentence should have been split in two, so a period has been added before “at,” and the word has been capitalized to begin a new sentence. In the corrected article, the legend now reads “24-Hour urine calcium through week 52. At week 52 of the PaTHway trial, overall median (IQR) 24-hour urine calcium excretion with TransCon PTH was 188.0 (103, 254) mg/day.”

In that same figure, the right side of the graph was not scaled appropriately to the figure's *y*-axis, and the ULN line was positioned incorrectly. The authors have provided a replacement for Figure 6 to address these errors, and the corrected figure is included below.

The article has been corrected online.

Original Figure 6:

**Figure:**
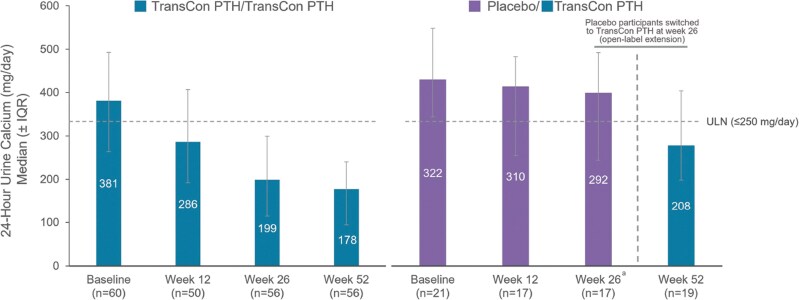


Corrected Figure 6:

**Figure:**
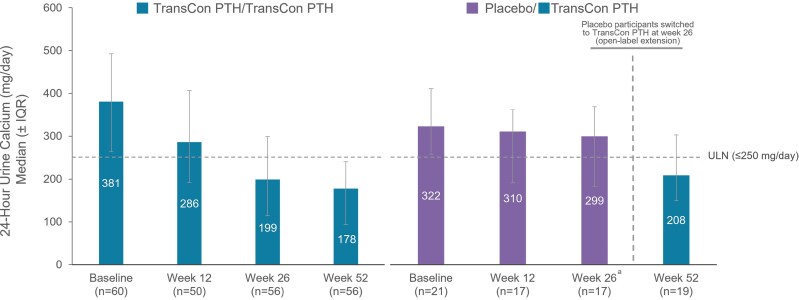


doi: 10.1210/clinem/dgae693

